# Olmesartan-Associated Enteropathy With Severe Diarrhoea and Acute Kidney Injury

**DOI:** 10.7759/cureus.104862

**Published:** 2026-03-08

**Authors:** Arina Nikolaeva, Andrea Della Vecchia

**Affiliations:** 1 Internal Medicine, Université Libre de Bruxelles, Brussels, BEL; 2 Internal Medicine, Hôpital Etterbeek-Ixelles, Brussels, BEL

**Keywords:** acute kidney injury, adverse drug effect, angiotension receptor blocker, chronic watery diarrhea, olmesartan-associated enteropathy, sprue-like enteropathy, villous atrophy

## Abstract

Olmesartan-associated enteropathy (OAE) is a rare but clinically significant adverse reaction to the angiotensin II receptor blocker (ARB) olmesartan. Clinically and histologically, it can mimic celiac disease but is typically reversible after drug discontinuation. We report the case of a 53-year-old man who presented with profuse watery diarrhea, dehydration, and acute kidney injury requiring hospitalization. An extensive diagnostic evaluation, including imaging studies, serological testing, and microbiologic assays, was negative. Duodenal biopsies demonstrated villous blunting with marked intraepithelial lymphocytosis. After discontinuation of olmesartan, gastrointestinal symptoms resolved completely, and renal function normalized within two weeks. Notably, an unintentional rechallenge with olmesartan led to rapid symptom recurrence, strongly supporting the diagnosis. This case highlights the need for a high clinical suspicion of OAE in patients with unexplained chronic diarrhea on olmesartan therapy. Early recognition is essential for recovery and helps avoid unnecessary invasive investigations and complications.

## Introduction

Olmesartan medoxomil is a widely prescribed angiotensin II receptor blocker (ARB) for the treatment of hypertension. Over the past decade, a serious gastrointestinal side effect, olmesartan-associated enteropathy (OAE), has been increasingly recognized since its first description in 2012 [1]. OAE presents with chronic diarrhea, weight loss, and villous atrophy on duodenal biopsy, thereby mimicking celiac disease. In contrast to celiac disease, OAE is not associated with gluten exposure, lacks celiac-specific serologic markers, and does not respond to a gluten-free diet [1]. 

The clinical challenge of OAE is its delayed onset, with symptoms often appearing months to years after drug initiation, making causality difficult to establish [2]. Misdiagnosis may result in extensive investigations and prolonged morbidity. Recognizing this enteropathy and promptly discontinuing olmesartan often leads to significant clinical improvement within weeks [2]. 

We describe a case of OAE in a 53-year-old man presenting with severe diarrhea, ARB, and acute kidney injury two years after initiating olmesartan. This case is notable for the severity of renal impairment, the unintentional drug rechallenge confirming the association, and complete recovery following drug cessation. The report emphasizes the importance of including OAE in the differential diagnosis of patients with unexplained gastrointestinal symptoms and ongoing use of olmesartan. 

## Case presentation

A 53-year-old man presented to the emergency department with a four-week history of profuse watery diarrhoea. The patient reported up to 50 episodes of diarrhoea per day during the most severe period, with associated nausea and vomiting developing over the three days before admission. The patient denied hematochezia, mucus in stools, fever, abdominal pain, or recent travel. There was no history of similar episodes in the past. 

His medical history was significant for hypertension, treated with a fixed-dose combination of olmesartan/amlodipine (20/5 mg daily) for the preceding two years. He reported no other medications and no known drug allergies and denied alcohol or illicit drug use. Family history was non-contributory for celiac disease or inflammatory bowel disease. 

The patient described a progressive decline over the weeks preceding admission, with significant fatigue and inability to maintain usual activities due to frequent diarrhoea. He estimated an unintentional weight loss of approximately 8 kilograms over four weeks, though no baseline weight was documented. 

On examination, he appeared fatigued and dehydrated, with dry mucous membranes, reduced skin turgor, and sunken eyes. Vital signs revealed a blood pressure of 112/72 mmHg, a heart rate of 94 beats/min, a respiratory rate of 18 breaths/min, a temperature of 36.8°C, and an oxygen saturation of 98% on room air. Cardiovascular and respiratory examinations were unremarkable. Abdominal examination revealed a soft, non-tender abdomen with normal bowel sounds and no organomegaly or palpable masses. There was no peripheral oedema. Neurological examination was normal. 

Investigations 

Initial laboratory results are summarised in Table 1. Findings included normocytic anaemia, leucocytosis, elevated C-reactive protein (CRP), and severe acute kidney injury with a prerenal profile. One year prior to admission, the patient's baseline creatinine was 1.1 mg/dL.

**Table 1:** The patient's laboratory results

Test Category	Parameter	Result	Reference Value	Interpretation
Haematology	Haemoglobin	11.5 g/dL	13.0–17.0 g/dL	Normocytic anemia
	White blood cell count	11.8 × 10³/μL	4.0–10.0 × 10³/μL	Leucocytosis
	C-reactive protein	53.5 mg/L	<5.0 mg/L	Elevated
Hepatic Function	Conjugated bilirubin	0.8 mg/dL	<0.3 mg/dL	Elevated
Renal Function	Glomerular filtration rate (Chronic Kidney Disease Epidemiology Collaboration)	7 mL/min/1.73m²	>90 mL/min/1.73m²	Severely reduced
	Serum creatinine	8.42 mg/dL	0.7–1.2 mg/dL	Markedly elevated
	Blood urea	129 mg/dL	7-20 mg/dL	Markedly elevated
Spot Urine Analysis	Urinary creatinine	139 mg/dL	39-259 mg/dL	
	Urinary sodium	40 mmol/L	71-171 mmol/L	
	Urinary osmolality	352 mOsm/kg		
Calculated Index	Fractional excretion of sodium (FENa)	0.3%		Prerenal acute kidney injury

An extensive infectious and autoimmune workup was negative, including a polymerase chain reaction (PCR) panel on stools for *Clostridioides difficile*, *Campylobacter *spp., *Salmonella *spp., *Shigella *spp., enteropathogenic *Escherichia coli*, and parasites; serological testing for HIV, antinuclear antibodies, anti-smooth muscle antibodies, antimitochondrial, and anti-tissue transglutaminase IgA and IgG antibodies. 

Given persistent diarrhoea and abnormal labs, a contrast-enhanced CT scan of the abdomen and pelvis was performed on day two of admission, revealing no evidence of colitis, bowel obstruction, masses, or lymphadenopathy.

**Figure 1:**
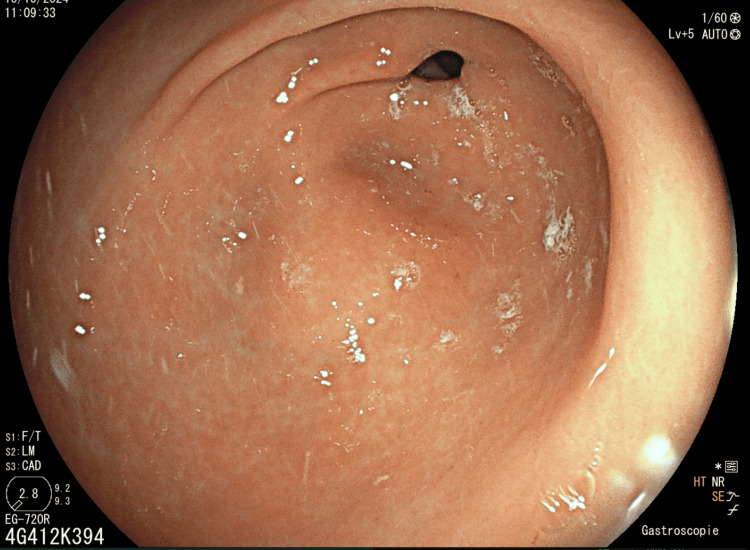
Erythematous and congestive appearance of the antrum

**Figure 2:**
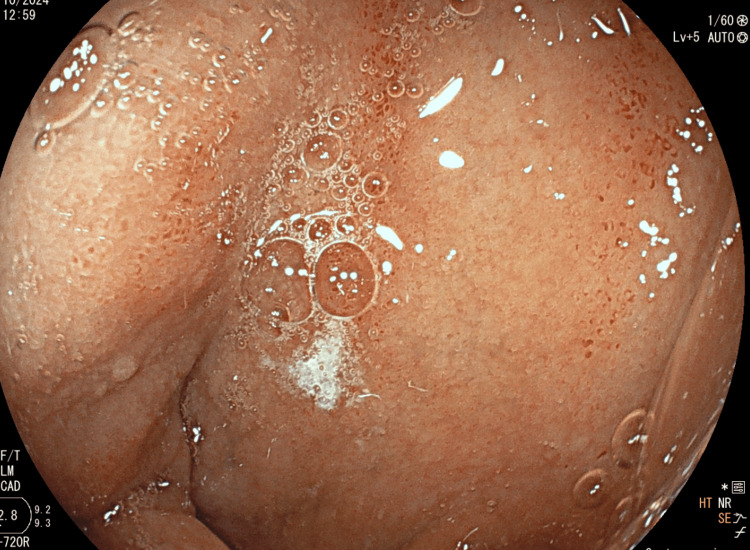
Petechial bulbitis observed on esophagogastroduodenoscopy

**Figure 3:**
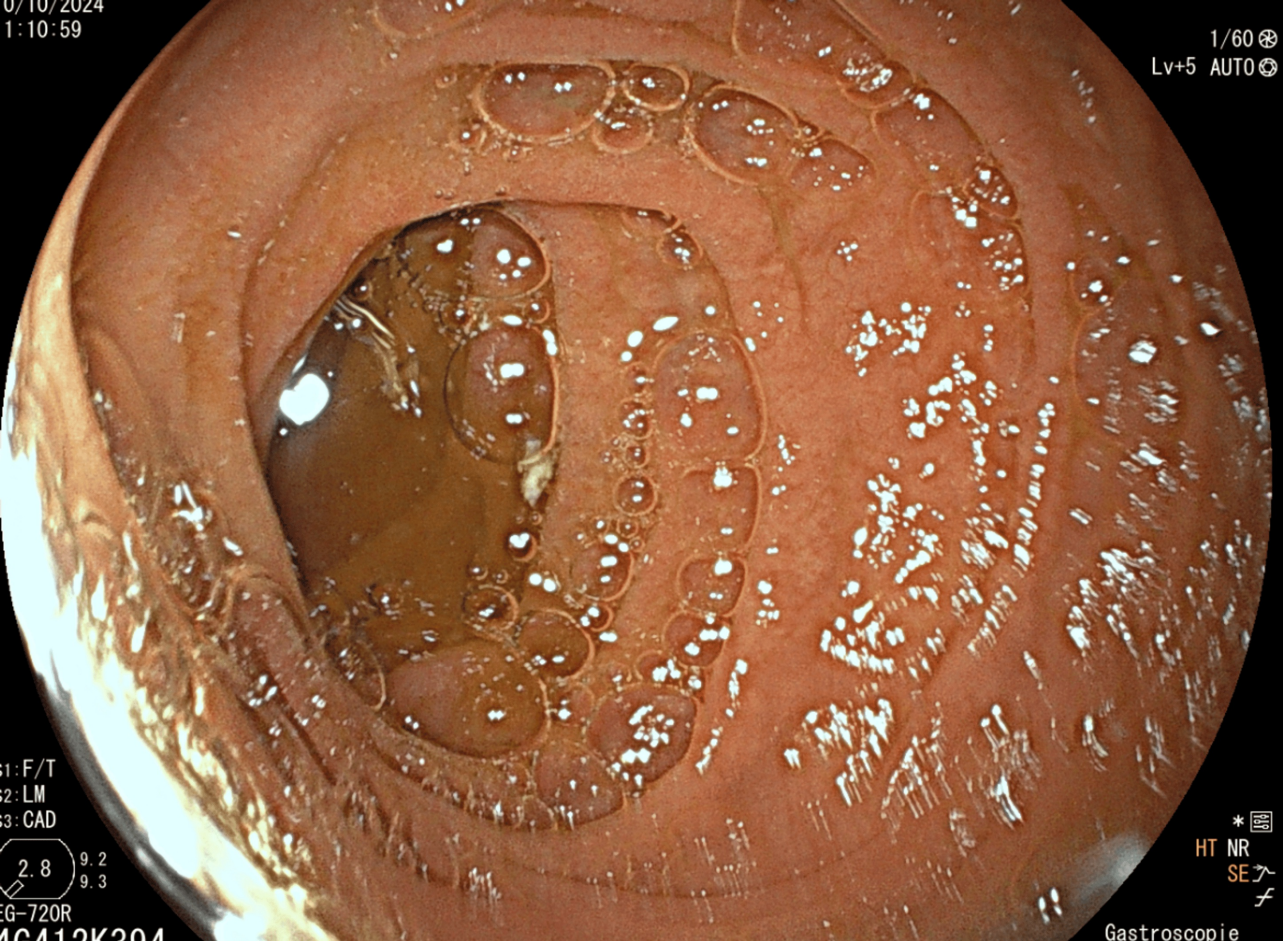
Clear visualization of microvilli and present valvulae conniventes

Esophagogastroduodenoscopy performed on day three showed Grade B oesophagitis according to the Los Angeles classification of gastroesophageal reflux disease [3], erythematous congestive gastropathy, and no macroscopic evidence of villous atrophy (Figures 1-3). Despite the normal gross appearance, multiple duodenal biopsies were obtained from the second part of the duodenum.

**Figure 4:**
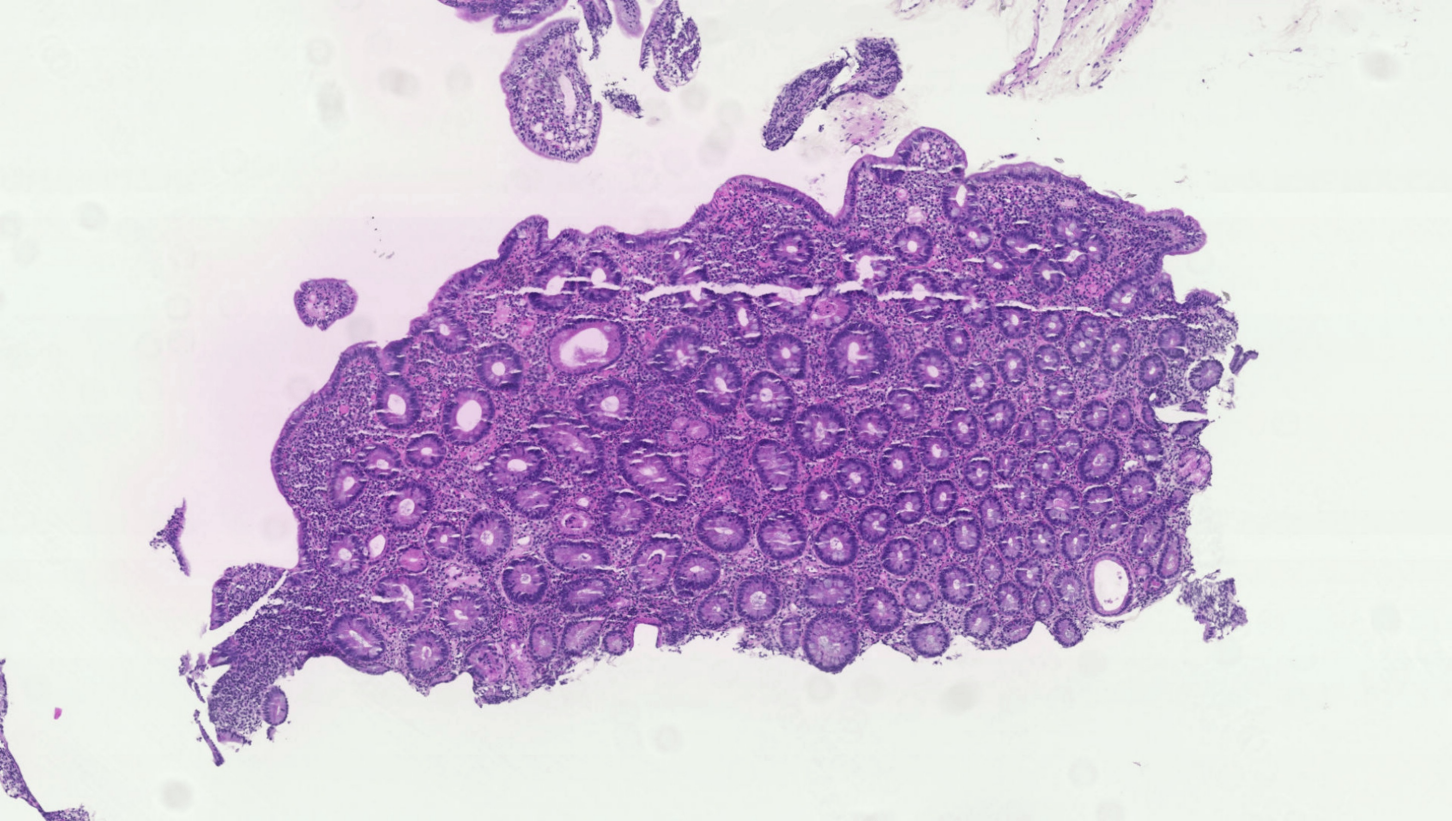
Duodenal mucosa with focal shortening and clubbing of the villi Covered by non-atypical epithelium infiltrated by a few lymphocytes and neutrophilic polymorphonuclear cells; the lamina propria is inflammatory with the presence of neutrophilic polymorphonuclear cells.

**Figure 5:**
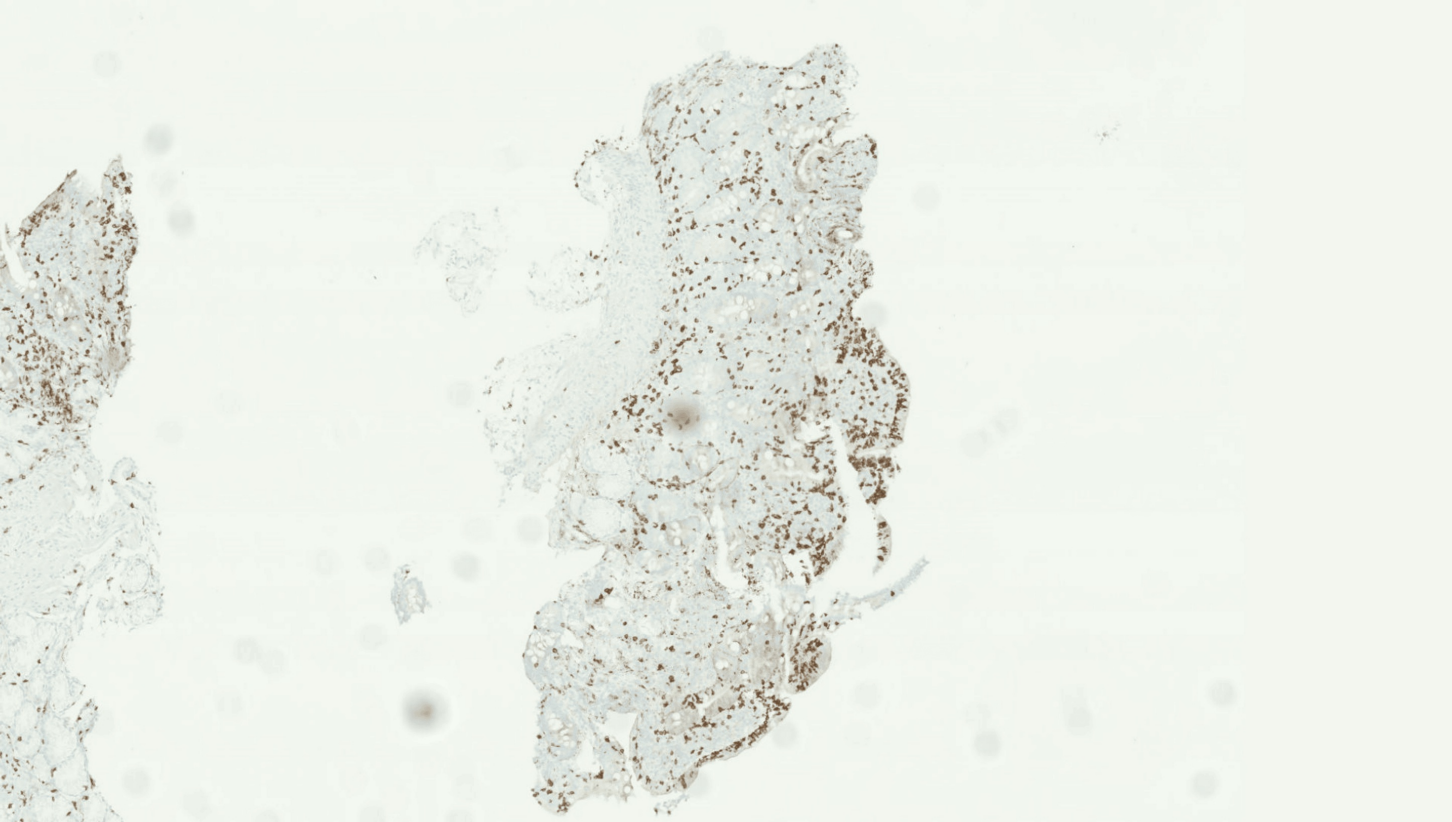
Immunohistochemical staining with anti-CD3 and anti-CD8 antibodies The anti-CD3 and CD8 immunostaining shows significant focal lymphocytic exocytosis.

Histopathological examination revealed focal villous blunting with increased intraepithelial lymphocytes and scattered neutrophil infiltration within the lamina propria (Figure 4). Immunohistochemical staining with anti-CD3 and anti-CD8 antibodies confirmed significant focal lymphocytic exocytosis (Figure 5), suggestive of an immune-mediated enteropathy. Although the clinical picture was consistent with a small bowel source, colonoscopy with biopsy was performed on day four to systematically exclude microscopic colitis, which can present with indistinguishable watery diarrhoea and requires histological diagnosis.

Differential diagnosis 

Based on the clinical presentation of profuse watery diarrhoea, significant weight loss, and severe dehydration causing prerenal acute kidney injury, we systematically evaluated potential aetiologies. 

Infectious causes were initially considered, given the acute severity, leucocytosis, and elevated CRP. However, comprehensive stool PCR testing for *Clostridioides difficile*, *Campylobacter*, *Salmonella*, *Shigella*, enteropathogenic *Escherichia coli*, and parasites returned negative. The absence of fever, haematochezia, recent antibiotic use, or travel history further excluded infectious aetiologies. 

Celiac disease was considered when duodenal biopsies revealed focal villous blunting with increased intraepithelial lymphocytes. However, negative anti-tissue transglutaminase IgA and IgG antibodies argued against this diagnosis. 

Inflammatory bowel disease, particularly Crohn's disease, was considered but seemed unlikely given the normal CT scan without bowel wall thickening or lymphadenopathy, normal gross endoscopic appearance, absence of granulomas on histopathology, and normal colonoscopy excluding microscopic colitis. 

Autoimmune enteropathy was raised given the CD3+/CD8+ lymphocytic infiltration, but a negative autoimmune serological panel made this diagnosis improbable. 

Common variable immunodeficiency (CVID) and immune dysregulation enteropathy were also considered. CVID can present with chronic diarrhoea, significant weight loss, and villous atrophy on biopsy in adulthood. The CVID-associated enteropathy is characterised by low serum immunoglobulin levels, impaired vaccine responses, and loss of plasma cells on biopsy. In our patient, serum immunoglobulins were within normal limits, and plasma cells were preserved on biopsy, excluding this diagnosis.

The diagnosis was found during a detailed medication review. The patient's two-year history of olmesartan therapy, combined with chronic severe diarrhoea with weight loss, villous atrophy mimicking celiac disease, negative celiac serology, negative infectious and autoimmune workup, and lack of improvement despite supportive measures while continuing olmesartan, raised a strong suspicion for olmesartan-associated enteropathy.

Treatment 

Olmesartan/amlodipine was discontinued upon establishing the diagnosis. The patient was switched to amlodipine 5 mg and perindopril 10 mg daily. He received intravenous isotonic saline rehydration throughout hospitalization, with monitoring of fluid balance and electrolytes. No specific treatment beyond drug cessation and supportive care was required. 

Outcome and follow-up

Within 48 hours of olmesartan discontinuation, the patient noted a marked reduction in diarrhea frequency. By day seven of hospitalization (two days after drug cessation), diarrhea had decreased to fewer than five episodes per day. The patient was discharged on day nine with complete resolution of gastrointestinal symptoms and significant improvement in renal function. At discharge, serum creatinine had decreased to 1.8 mg/dL with an estimated glomerular filtration rate (eGFR) of 42 mL/min/1.73 m². 

Follow-up laboratory testing performed 14 days after olmesartan discontinuation showed normalization of renal function (serum creatinine 1.1 mg/dL, eGFR 78 mL/min/1.73 m²) and resolution of anemia (hemoglobin 13.2 g/dL) and inflammatory markers (CRP 4.2 mg/L). 

At an outpatient follow-up visit scheduled three weeks after discharge, the patient reported that he had independently restarted olmesartan several days earlier, believing symptoms had been due to a transient infection. Within 48 hours of restarting olmesartan, he experienced a recurrence of profuse watery diarrhea with more than 25 episodes per day. He contacted our department and was instructed to stop olmesartan immediately. Symptoms resolved completely within 72 hours of the second discontinuation. This unintentional rechallenge strongly supported olmesartan as the causative agent. The patient has remained symptom-free on amlodipine/perindopril therapy. A repeat esophagogastroduodenoscopy with duodenal biopsies was not performed during the follow-up period, as the patient achieved complete symptomatic resolution and normalization of laboratory parameters. This represents a limitation of the current report, as histological recovery cannot be confirmed. A clinical timeline is provided in Table 2.

**Table 2:** Clinical timeline of olmesartan-associated enteropathy from drug initiation through unintentional rechallenge to sustained remission

Phase	Time Period	Key Events
Drug Initiation	Month 0	Olmesartan/amlodipine 20/5 mg started for hypertension
Latency Period	Months 1-22	Asymptomatic period on olmesartan therapy
Symptom Development	Month 23	Progressive severe diarrhea (up to 50 episodes/day), 8 kg weight loss
Hospitalization	Day 0	Admission with severe dehydration and acute kidney injury
Diagnostic Phase	Days 1-5	Negative infectious/autoimmune workup; duodenal biopsy showed villous atrophy with lymphocytosis; OAE diagnosis established
Treatment	Day 5	Olmesartan discontinued; switched to amlodipine/perindopril
Discharge	Days 9	Rapid symptom resolution; improvement in renal function
Unintentional Rechallenge	Day 25	Patient independently restarted olmesartan
Recurrence	Day 27	Diarrhea recurred within 24 hours (>30 episodes/day)
Final Cessation	Day 27	Olmesartan permanently discontinued
Second Recovery	Day 30	Complete resolution within 72 hours
Follow-up	Month 4	Sustained remission on amlodipine/perindopril

## Discussion

This case illustrates a severe manifestation of OAE complicated by acute kidney injury requiring hospitalization. The unintentional drug rechallenge with rapid symptom recurrence provided pharmacological evidence for causal association.

Olmesartan medoxomil is an ARB that selectively blocks the angiotensin II type 1 receptor, lowering peripheral vascular resistance [4]. It is effective for hypertension but is particularly linked to a sprue-like enteropathy, first systematically described by Rubio-Tapia et al. in 2012 [1]. Their series of 22 patients defined key features: chronic diarrhea, significant weight loss, and villous atrophy on biopsy with complete resolution after drug discontinuation [1]. Following accumulating evidence, the U.S. Food and Drug Administration issued a safety communication in 2013, requiring label modifications about sprue-like enteropathy risk [5]. Despite regulatory action, OAE remains underdiagnosed in clinical practice, likely due to its variable latency period and phenotypic overlap with more prevalent causes of chronic diarrhea.

A systematic review by Ianiro et al. (2014) identified 54 cases of OAE with a latency period between drug initiation and symptom onset between six months and seven years [2]. Our patient's presentation after two years of olmesartan falls within this timeframe. Common laboratory abnormalities included normocytic anemia (45% of patients) and hypoalbuminemia (39%) [2]. All patients who discontinued olmesartan experienced complete resolution of diarrhea, demonstrating the reversible nature of this condition [2].

A multi-database cohort study by Dong et al. (2018) investigated the association between olmesartan and enteropathy, including nearly two million patients, comparing olmesartan with other ARBs (valsartan and losartan) [6]. The analysis demonstrated statistically significant hazard ratios for olmesartan: 1.21 for celiac disease diagnosis, 1.22 for diarrhea accompanied by weight loss, and 1.04 for non-infectious enteropathy [6]. While absolute incidence remained low, this provided robust epidemiological support for a drug-specific effect distinct from class-wide ARB toxicity.

OAE histology includes variable villous atrophy, increased intraepithelial lymphocytes, chronic lamina propria inflammation, and occasional subepithelial collagen deposition. Mauloni et al. observed villous atrophy in 93.5% of cases, with 61% showing total villous atrophy and 32.4% demonstrating partial atrophy [7]. These microscopic changes closely mimic celiac disease, creating diagnostic challenges. In our patient, the duodenal mucosa appeared macroscopically normal, yet biopsies revealed focal villous blunting with intraepithelial lymphocytic infiltration. This dissociation emphasizes obtaining duodenal biopsies in suspected enteropathy despite an unremarkable endoscopy. Immunohistochemistry demonstrating CD3+ and CD8+ T-lymphocyte predominance further supported an immune-mediated process. Colonoscopy with biopsy was additionally performed to systematically exclude microscopic colitis, which can present with clinically indistinguishable watery diarrhea and requires histological diagnosis to reliably exclude.

Similar histological patterns may occur with other ARBs, though far less commonly than with olmesartan, and with certain immunosuppressive medications, including methotrexate, mycophenolate mofetil, and azathioprine [7], underlining the need for thorough medication review.

While most OAE cases resolve without sequelae, severe complications can occur. Our patient's acute kidney injury represents a severe clinical presentation documented in the OAE literature. Prerenal azotemia reflected extreme fluid losses from profuse diarrhea. Complete renal function normalization within 14 days of olmesartan withdrawal and adequate hydration demonstrates reversibility of this complication when promptly managed. Beyond renal complications, other severe sequelae have been reported. Tan et al. described a case of OAE complicated by acute ischemic hepatitis, manifesting as marked transaminase elevation that normalized following olmesartan withdrawal [8]. Moreover, Alexander et al. reported jejunal ischemia progressing to bowel perforation requiring emergency surgical intervention, with histopathological examination confirming OAE [9]. These cases emphasize the importance of early diagnosis. 

Regarding antihypertensive management following olmesartan withdrawal, current evidence suggests that OAE represents a drug-specific phenomenon rather than a class effect [10]. In a French nationwide cohort study, olmesartan increased hospitalization for intestinal malabsorption and showed a strong duration-response compared to other ARBs [10]. This supports the absence of meaningful class-wide cross-reactivity. In our patient, substitution with perindopril and amlodipine was well tolerated, with no recurrence of gastrointestinal symptoms at four months of follow-up. Where an ARB is clinically preferred, available evidence suggests that switching to a non-olmesartan ARB is likely safe, though close monitoring for symptom recurrence is advisable.

Pathophysiological mechanisms of olmesartan-induced enteropathy remain incompletely elucidated. Current evidence suggests dysregulation of intestinal mucosal immunity as a central mechanism. Olmesartan may trigger activation of cell-mediated immune pathways, leading to T-lymphocyte infiltration and epithelial injury. Additionally, inhibition of transforming growth factor-β (TGF-β) signaling has been implicated, as TGF-β maintains immune homeostasis and epithelial barrier integrity [11]. Genetic susceptibility likely modulates the risk of OAE. Higher HLA-DQ2 prevalence has been reported in affected patients (approximately 68%) versus the general population (25%-30%) [11]. Although this mirrors the HLA association seen in coeliac disease, OAE remains clinically distinct: patients typically have negative coeliac serology and do not improve on a gluten-free diet. These features support OAE as a drug-specific enteropathy rather than latent celiac disease. The variability in clinical severity observed across reported cases, ranging from mild diarrhea to life-threatening dehydration, as in our patient, may in part reflect pharmacogenomic differences in olmesartan metabolism or intestinal immune responsiveness, though this requires further investigation.

## Conclusions

OAE remains an underrecognized cause of chronic diarrhea that can lead to severe complications if diagnosis is delayed. Clinicians should suspect this condition in any patient on olmesartan therapy who presents with unexplained diarrhea, particularly when initial investigations for infectious and autoimmune etiologies are negative. The key to diagnosis lies in obtaining duodenal biopsies even when the mucosa appears endoscopically normal, as histopathological findings may be the only diagnostic clue. Once suspected, immediate olmesartan discontinuation is both diagnostic and therapeutic, with complete symptom resolution within days to weeks. The potential for severe systemic complications, including life-threatening dehydration and acute kidney injury, highlights the importance of early recognition and prompt drug withdrawal. Given the widespread prescription of olmesartan and the reversible nature of this adverse effect with drug substitution, increased awareness among clinicians is essential. Early recognition can prevent unnecessary invasive investigations and patient suffering, as well as reduce healthcare costs.

## References

[1] Rubio-Tapia A, Herman ML, Ludvigsson JF, Kelly DG, Mangan TF, Wu TT, Murray JA (2012). Severe spruelike enteropathy associated with olmesartan. Mayo Clin Proc.

[2] Ianiro G, Bibbò S, Montalto M, Ricci R, Gasbarrini A, Cammarota G (2014). Systematic review: sprue-like enteropathy associated with olmesartan. Aliment Pharmacol Ther.

[3] Sami S, Ragunath K (2013). The Los Angeles classification of gastroesophageal reflux disease. Video J Encyc GI Endosc.

[4] Al-Majed AA, Bakheit AH, Abdel Aziz HA, Al-Jallal AA (2017). Olmesartan. Profiles Drug Subst Excip Relat Methodol.

[5] (2026). FDA Drug Safety Communication: FDA approves label changes to include intestinal problems (sprue-like enteropathy) linked to blood pressure medicine olmesartan medoxomil. https://www.fda.gov/drugs/drug-safety-and-availability/fda-drug-safety-communication-fda-approves-label-changes-include-intestinal-problems-sprue.

[6] Dong YH, Jin Y, Tsacogianis TN, He M, Hsieh PH, Gagne JJ (2018). Use of olmesartan and enteropathy outcomes: a multi-database study. Aliment Pharmacol Ther.

[7] Mauloni PA, Capuani F, Paone C (2021). An unusual cause of diarrhoea: case report and literature review of olmesartan-associated enteropathy. Eur J Gastroenterol Hepatol.

[8] Tan R, Abasszade JH, Dhillon H, Kuan CC, Worland T, Tabatabai S (2024). Severe hepatitis complicating olmesartan enteropathy: a case report. Case Rep Gastroenterol.

[9] Alexander D, Abdelazeem B, Alnounou M (2023). Olmesartan-induced ischemic enteritis complicated with bowel perforation: a case report and literature review. Cureus.

[10] Basson M, Mezzarobba M, Weill A, Ricordeau P, Allemand H, Alla F, Carbonnel F (2016). Severe intestinal malabsorption associated with olmesartan: a French nationwide observational cohort study. Gut.

[11] Galanopoulos M, Varytimiadis L, Tsigaridas A (2017). Small bowel enteropathy associated with olmesartan medoxomil treatment. Ann Gastroenterol.

